# A Sensitive and Selective Liquid Chromatography/Tandem Mass Spectrometry Method for Quantitative Analysis of Efavirenz in Human Plasma

**DOI:** 10.1371/journal.pone.0063305

**Published:** 2013-06-05

**Authors:** Praveen Srivastava, Ganesh S. Moorthy, Robert Gross, Jeffrey S. Barrett

**Affiliations:** 1 Division of Clinical Pharmacology & Therapeutics, The Children's Hospital of Philadelphia, Philadelphia, Pennsylvania, United States of America; 2 Departments of Pediatrics, Medicine and Biostatistics and Epidemiology, University of Pennsylvania, Perelman School of Medicine, Philadelphia, Pennsylvania, United States of America; University of Pittsburgh, United States of America

## Abstract

A selective and a highly sensitive method for the determination of the non-nucleoside reverse transcriptase inhibitor (NNRTI), efavirenz, in human plasma has been developed and fully validated based on high performance liquid chromatography tandem mass spectrometry (LC–MS/MS). Sample preparation involved protein precipitation followed by one to one dilution with water. The analyte, efavirenz was separated by high performance liquid chromatography and detected with tandem mass spectrometry in negative ionization mode with multiple reaction monitoring. Efavirenz and ^13^C_6_-efavirenz (Internal Standard), respectively, were detected via the following MRM transitions: m/z 314.20243.90 and m/z 320.20249.90. A gradient program was used to elute the analytes using 0.1% formic acid in water and 0.1% formic acid in acetonitrile as mobile phase solvents, at a flow-rate of 0.3 mL/min. The total run time was 5 min and the retention times for the internal standard (^13^C_6_-efavirenz) and efavirenz was approximately 2.6 min. The calibration curves showed linearity (coefficient of regression, *r*>0.99) over the concentration range of 1.0–2,500 ng/mL. The intraday precision based on the standard deviation of replicates of lower limit of quantification (LLOQ) was 9.24% and for quality control (QC) samples ranged from 2.41% to 6.42% and with accuracy from 112% and 100–111% for LLOQ and QC samples. The inter day precision was 12.3% and 3.03–9.18% for LLOQ and quality controls samples, and the accuracy was 108% and 95.2–108% for LLOQ and QC samples. Stability studies showed that efavirenz was stable during the expected conditions for sample preparation and storage. The lower limit of quantification for efavirenz was 1 ng/mL. The analytical method showed excellent sensitivity, precision, and accuracy. This method is robust and is being successfully applied for therapeutic drug monitoring and pharmacokinetic studies in HIV-infected patients.

## Introduction

Efavirenz is a non-nucleoside reverse transcriptase inhibitor (NNRTI) used in the treatment of HIV-1 infection in combination with other anti-retroviral agents in children and adults. NNRTIs stereo-tactically inhibit the target enzyme and suppress viral replication with attendant immunological benefits. NNRTIs bind directly and reversibly to the catalytic site of the reverse transcriptase enzyme and therefore, interfere with viral RNA to DNA-directed polymerase activities [1], [2]. Several analytical methods have been developed to quantify NNRTI, in human plasma, but in many cases HPLC with ultraviolet (UV) detection [3]–[20], LC-MS [21]–[24], and HPLC with fluorescence detection [25], and capillary electrophoresis [26] were used to quantify antiretroviral drugs. Samples volumes range from 0.2–0.3 mL plasma [3], [4], [5], [8]–[12], although smaller sample volumes (0.1 mL) have been used successfully [5]. HPLC/UV- based methods aim at simultaneous quantification of several drugs, but has limited selectivity and sensitivity. This requires a relatively long run time and high sample volume. In contrast, LC-MS/MS techniques are preferred for their shorter runtimes, and high selectivity and sensitivity. Different sample preparation methods, such as liquid-liquid extraction [4], [5], [8] solid phase extraction [3], [9]–[12], and protein precipitation followed by dilution [5] have been used to extract efavirenz from plasma. Besides, all methods use a complicated time-consuming sample pre-treatment consisting of solid-phase or liquid–liquid extraction combined with evaporation of the extract to dryness, which is subsequently reconstituted. Furthermore, complicated instrument set-up is necessary consisting of a combination of two HPLC systems. In the present investigation, we report a rapid, selective, sensitive, reproducible and cost effective bioanalytical method for the determination of efavirenz in human plasma. We utilized a sample volume of 50 μL with ^13^C-labeled internal standard (^13^C_6_-Efavirenz) and LLOQ of 1.0 ng/mL. Other reported methods used commercially available internal standards (propanolol), and an LLOQ of 20 ng/mL was reported [24]. The analytical method is fully validated in accordance with FDA guidelines [27], using LC-MS/MS tandem mass spectrometry.

The objective of this investigation was to develop and validate a simple, selective and sensitive LC-MS/MS method for the quantification of efavirenz in human plasma to support an early phase pharmacokinetic study of efavirenz in patients. Although previous assays for other NNRTI have been reported, to our knowledge this is the first method developed for the detection of efavirenz in human plasma with an LLOQ of 1 ng/mL and short analytical run time of 5 min for the fast analysis of samples for monitoring of efavirenz in human plasma samples.

## Experimental

### Reagent and chemicals

Efavirenz ((S)-6-chloro-4-(cyclopropylethynyl)-l,4-dihydro-4-(trifluoromethyl)-2H-3,l-benzoxazin-2-one) and internal standard, ^13^C_6_-efavirenz were purchased from Alsachim (Strasbourg, France) with purity of 99.0% and 99.3% respectively. Different lots of drug-free (blank) human plasma were obtained from the blood bank at The Children's Hospital of Philadelphia. HPLC-grade acetonitrile was purchased from Fisher-Scientific (Pittsburgh, PA, USA) and reagent-grade formic acid (∼96%) was purchased from Sigma-Aldrich Co. (St. Louis, MO, USA). Deionized water was prepared using a Nanopure water purifying system from Thermo Fischer Scientific.

### Liquid chromatography

The Shimadzu HPLC system consisted of two LC-20AD delivery pumps, a DGU-20A5 Shimadzu vacuum degasser, a SIL-20AC Shimadzu auto sampler and a CBM-20A system controller (Shimadzu Scientific Instruments; Columbia, MD, USA). HPLC separations were performed on a Waters Xbridge C18 analytical column (50 mm×2.1 mm, 3.5 µm 120 Å). Mobile phase A consisted of water with 0.1% formic acid and mobile phase B consisted of acetonitrile with 0.1% formic acid. The gradient was as follows: 0.0–1.5 minutes mobile phase A 98% to 2%, with divert valve off; 1.51–3.00 minutes mobile phase A 2%, mobile phase B 98%; 3.1–5 minutes mobile phase A 98%. The flow rate was 0.3 mL/min and 10 µL of the sample was injected for each analysis. The column and auto sampler were maintained at 40°C and 15°C, respectively. An electronic valve actuator with a Rheodyne selector valve was used to divert LC flow to waste, at the first 1 minute, when no data acquisition was taking place.

### Mass spectrometry analysis

Samples were analyzed with an AB Sciex 4000 triple quadropole mass spectrometer equipped with Turbo Ionspray. Analyst version 1.4.2 software (MDS Sciex; Toronto, Canada) was used to control this equipment and for acquiring and processing data. The negative ion mode for MS/MS analysis was selected as it gave the maximum response. Nitrogen was used as the nebulizer, auxiliary, collision and curtain gases. Analytes were detected by tandem mass spectrometry using multiple reaction monitoring (MRM) with a dwell time of 250 ms. For the determination of the precursor and product of ion spectra a solution of 1 µg/mL efavirenz or internal standard in mobile phase was infused directly into the ion sources with a Harvard Apparatus syringe pump at a flow rate of 10 µL/min. The most intense precursor-to-fragment transitions using negative turbo spray were: efavirenz, m/z 314.20→243.90; ^13^C_6_-efavirenz (Internal standard) 320.20→249.90.

The conditions for ionization of efavirenz and internal standard were optimized using individual standard solutions, each at 100 ng/mL, which were infused by a syringe pump through a Tee device at a flow rate of 10 µL/min into the stream of mobile phase eluting from the LC column through a mixing Tee and then into the turbo spray source, to mimic the LC-MS/MS conditions. The main working parameters of the mass spectrometer were: collision activated dissociation (CAD) gas, medium; curtain gas, 35; Gas 1 (nebulizer gas) 50; Gas 2 (heater gas) 30; source temperature 550°C. The optimized declustering potential (DP), entrance potential (EP), collision energy (CE), and collision cell exit potential (CXP) were set at −55.40, −7.0, −25.3, −4.64 for Efavirenz, and −53.24, −10, −25.11, −4.39 for ^13^C_6_-efavirenz (Internal standard), respectively.

### Preparation of standards and quality control (QC) samples

Two independent primary stock solutions of efavirenz were prepared by dissolving efavirenz in 100% acetonitrile producing a concentration of 1 mg/mL and were stored at –20°C. The concentrations of efavirenz in two stock solutions were compared and the relative percent deviation between two stocks was <5%. The primary stock solution was diluted in acetonitrile to prepare two intermediate stock solutions of 50 µg/mL which are used to prepare standards and QCs in plasma. Ten standards containing efavirenz concentrations of 1, 2.5, 5, 10, 50, 100, 250, 500, 1000 and 2500 ng/mL were prepared by serial dilution from the first intermediate stock in human plasma. Four QCs were prepared at 4, 80, 800 and 2000 ng/mL, representing low (4 ng/mL), medium (80 and 800 ng/mL) and high QCs (2000 ng/mL). High QC (2000 ng/mL) was prepared in human plasma by spiking Efavirenz stock solution (50 µg/mL). High QC (2000 ng/mL) was serially diluted with human plasma to provide medium QCs (80 and 800 ng/mL) and low (4 ng/mL) QCs. Drug free blank human plasma lots were used for bulk preparation of standards and QCs. Selectivity is ensured at LLOQ in 6 different lots of human plasma plus one pooled blank human plasma lots. The internal standard stock solution (1 mg/mL) was prepared by dissolving ^13^C_6_-efavirenz in 1% formic acid in acetonitrile. The internal standard working solution was prepared by diluting the internal standard stock solution with 0.1% formic acid in acetonitrile into a single working solution with a final concentration of 10 ng/mL. Amber glass vials were used for storing stock solutions.

### Sample preparation

Internal standard solution (150 µL, 10 ng/mL) in acetonitrile was added to 50 μL of blank, standards, QCs and unknown samples in a 96 well plate. The plate was vortex-mixed on 96 well plate-mixer at 500 rpm for 10 minutes and centrifuged at 3500 rpm for 10 minutes. A volume of 70 μL of the supernatant was aliquoted into fresh clean 96-well plate and diluted with 70 μL of milli-Q water. Diluted supernatant was vortex-mixed at 500 rpm for 10 minutes and centrifuged at 3500 rpm for 10 minutes and injected into the LC-MS/MS system, with an injection volume of 10 μL.

### Method validation

Method validation was performed according to guidelines set by the United States Food and Drug Administration (FDA) for bioanalytical method validation [27]. This method was validated in terms of linearity, specificity, low limit of quantitation (LLOQ), recovery, intra-day and inter-day accuracy and precision, carryover assessment, dilution integrity, matrix effect, and stability of the analyte during sample storage and processing procedures. Each analytical run included a double blank sample (human plasma without internal standard), a blank sample (human plasma with internal standard), ten standard concentrations for calibration, and replicate sets of QC samples: LLOQ QC 1 ng/mL (used for three core validation runs), low QC (LQC) 4 ng/mL, medium QCs (MQC) 80 ng/mL and 800 ng/mL, and high QC (HQC) 2000 ng/mL. LLOQ QC (1 ng/mL) was used for 3 core validation runs to demonstrate method accuracy and precision at LLOQ concentration. Six sets of QCs were used during 3 core validation runs, for assessing intra-day and inter-day accuracy and precision. For stability studies and sample analysis only low, medium and high QCs are used for accepting the batch.

### Linearity and sensitivity

For the evaluation of the linearity of the standard calibration curve, the analyses of efavirenz in plasma samples were performed on three independent days using fresh preparations. The calibrations curves were prepared over a linear range of 1–2500 ng/mL at ten concentrations of 1, 2.5, 5, 10, 50, 100, 250, 500, 1000 and 2500 ng/mL. Each calibration curve consisted of a double blank sample, a blank sample and ten calibrator concentrations. Another two double blank samples were analyzed immediately following the highest concentration standard in each run to monitor for carry-over of efavirenz or the internal standard. Carryover was also assessed by quantification of two LLOQ (1 ng/mL) samples after injecting ULOQ (2500 ng/mL).

The calibration curve was developed using the following criteria: (1) the mean value should be within ±15% of the theoretical value, except at the LLOQ, where it should not deviate by more than ±20%; (2) the precision around the mean value should not exceed a 15% coefficient of variation (CV), except for LLOQ, where it should not exceed a 20% CV; (3) at least 75% of the non-zero standards of each nominal concentration should meet the above criteria; (4) the correlation coefficient (r) should be greater than or equal to 0.99.

Each calibration curve was constructed by plotting the analyte to internal standard peak area ratio (y) against the analyte concentrations (x). The calibration curves were fitted using a least-square linear regression model *y  =  ax+b*, weighted by none, 1/×, 1*/x^2^* using the Analyst ^®^ software. The resulting a, b and c parameters were used to determine back-calculated concentrations, which were then statistically evaluated.

### Specificity

The specificity was defined as non-interference at retention times of efavirenz from the endogenous plasma components and no cross-interference between efavirenz and internal standard using the proposed extraction procedure and LC-MS/MS conditions. Six different lots of blank (efavirenz free plasma) were evaluated with and without internal standard to assess the specificity of the method at LLOQ QC. Potential interference by antiretroviral drugs (tenofovir, emtricitabine, and ribavirin) concomitantly administered to the patients was also evaluated by spiking blank plasma at their therapeutic concentrations. An “interfering drug” has been considered as a molecule which exhibits a retention time close to 0.3 min from the analytes, and with the potential capability to cause ion suppression or enhancement.

### Accuracy and precision

The intra- and inter-assay precisions were determined using the CV (%) and the intra- and inter-assay accuracies were expressed as the percent difference between the measured concentration and the nominal concentration. The % accuracy of the method was expressed by the formula: % accuracy =  (measured concentration/nominal concentration) ×100. Intra-assay precision and accuracy were calculated using replicate (n = 6) determinations for each concentration of the spiked plasma sample during a single analytical run. Inter-assay precision and accuracy were calculated using replicate (n = 6) determination of each concentration made on three separate days.

### Recovery (extraction efficiency) and matrix effect

The extraction efficiency of efavirenz was determined by analyzing six replicates of efavirenz plasma samples at four QC concentrations: low QC (4 ng/mL), medium QCs (80, and 800 ng/mL) and high QC (2000 ng/mL). Recovery was calculated by comparing the peak areas of efavirenz added into blank plasma and extracted using the protein precipitation procedure with those obtained from efavirenz spiked directly into diluted blank post extraction solvent at the four QC concentrations. The matrix effect was measured by comparing the peak response of the post-extracted spiked sample at low QC (4 ng/mL) with those of the pure standards containing equivalent amounts of the efavirenz prepared in neat solution (50% acetonitrile).

### Stability study

The stability of efavirenz in human plasma was assessed by analyzing replicate QC samples (n = 6) at concentrations of low QC (4 ng/mL), medium QCs (80, and 800 ng/mL) and high QC (2000 ng/mL), during the sample and storage procedure. For all stability studies, freshly prepared and stability testing QC samples were evaluated by using a freshly prepared standard curve for the measurement. The short term (bench top) stability was assessed at low QC (4 ng/mL), and high QC (2000 ng/mL), after exposure of the plasma samples to room temperature for 4 hours (data not shown) and 24 hours, respectively. The freeze/thaw stability was determined after three freeze/thaw cycles (room temperature to −80°C). The freeze/thaw stability is also assessed at low QC (4 ng/mL), and high QC (2000 ng/mL). Autosampler stability was determined by letting low QC (4 ng/mL), medium QCs (80, and 800 ng/mL) and high QC (2000 ng/mL) sit in autosampler for 72 hrs. The concentrations obtained from all stability studies were compared to freshly prepared QC samples, and the percentage concentration deviation was calculated. The analytes were considered stable in human plasma when the concentration difference was less than 15% between the freshly prepared samples and the stability testing samples.

## Results

### Linearity and sensitivity

**Figure 1:**
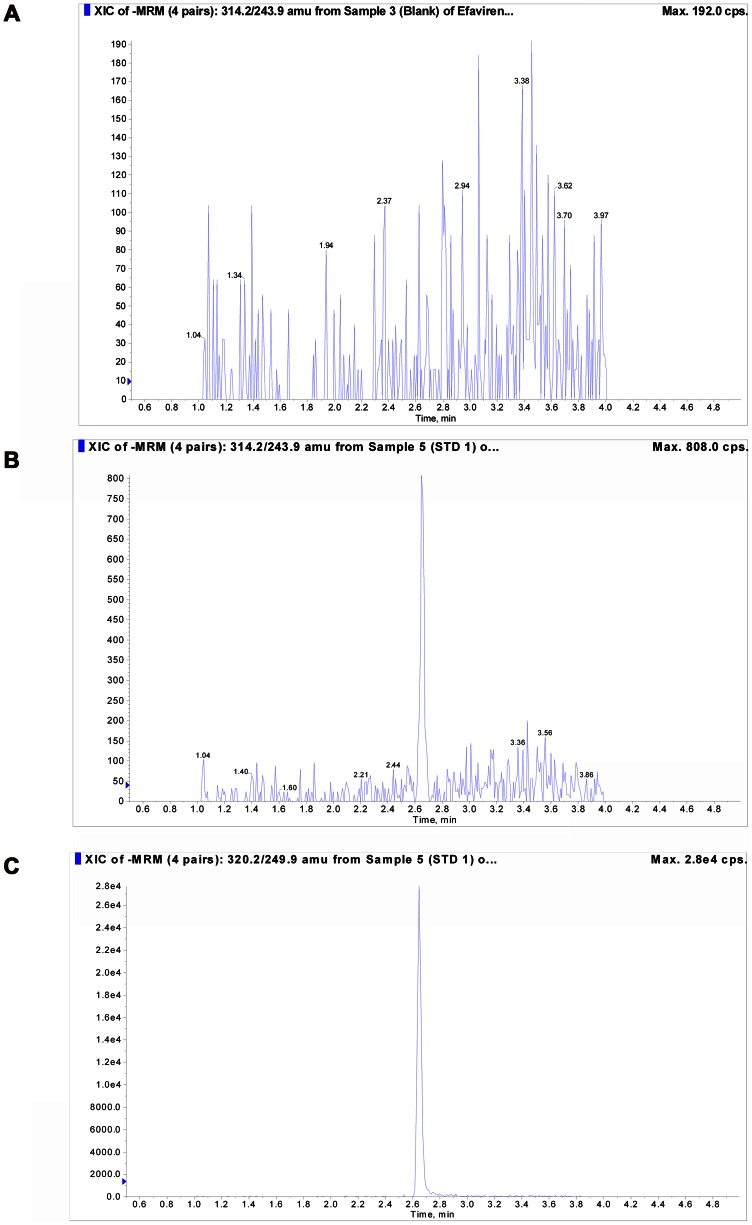
Representative chromatograms. A) Blank human plasma, B) Efavirenz in human plasma at Lowest limit of quantification (LLOQ, 1.0 ng/mL) and C) ^13^C_6_-Efavirenz (Internal standard, 10 ng/mL) in human plasma.

**Figure 2:**
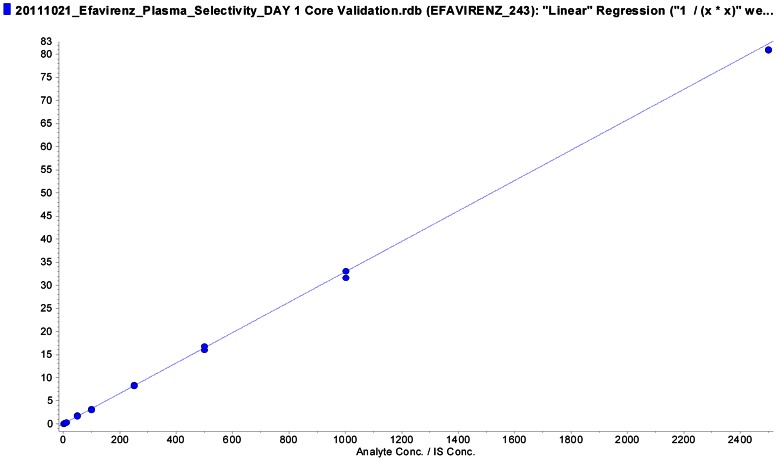
Calibration Curve of Efavirenz in human plasma (1.0 ng/mL, r = 0.9984).

The method was validated under the above criteria and found to be linear from concentrations of 1 to 2,500 ng/mL. The correlation coefficient (r) from inter-day analysis was >0.99 in all cases. The calibration model was selected based on the analysis of the data by linear regression with or without weighing factors (none, 1/× and 1/×^2^). The best linear fit and least-square residuals for the calibration curve were achieved with a 1/×^2^ weighing factor, giving a representative mean linear regression equation for the calibration curve of efavirenz: Y =  0.0329 (±0.0003)× +0.00638 (±0.00100). Y represents the peak area ratio of the analyte to the internal standard and x is the concentration of the analyte. The mean correlation coefficient of the weighted calibration curve generated during the three day validation for efavirenz using various weighing are as shown below. Weighing (none): r = 0.9990 (±0.0010); Weighing (1/×): r = 0.9981 (±0.0005) and Weighing (1/×^2^): r = 0.9990 (±0.0003). Based on these determinations, we chose 1/×^2^ as weighing factor for linear regression. The sensitivity at LLOQ (1 ng/mL) for the method was assessed in 6 different lots of blank human plasma and pooled human plasma, demonstrating a %CV of less than 8% (precision) and an accuracy in the range of 80–105%, with a signal-to-noise (S/N) ratio of >7. Representative chromatogram of blank, LLOQ, internal standard are shown in Figure 1 and a representative calibration curve is shown in Figure 2.

### Carryover assessment

Carryover was evaluated on each run during the validation. Two carryover double blanks were analyzed immediately following Upper Limit of Quantification (ULOQ) samples in each run. The carryover of efavirenz was 0.01% with first double blank and no carryover was observed in the second double blank placed after ULOQ. Carryover was also assessed by quantification of two LLOQ (1 ng/mL) samples after injecting ULOQ (2500 ng/mL). It was found that accuracy of the first LLOQ sample was 126% and second LLOQ sample was 108%.

### Precision and Accuracy

At the ten calibration standards, the inter-day precision ranged from 4.38-11.1% and the accuracy ranged from 96.1–104% (Table 1). The intraday precision based on the standard deviation of replicates of lower limit of quantification (LLOQ) was 9.24% and for QC samples ranged from 2.41 to 6.42% and with accuracy of 112% and 100–111% for LLOQ and QC samples. The inter day precision was 12.3% and 3.03–9.18% for LLOQ and QC samples, and the accuracy was 108% and 95.2–108% for LLOQ and QC samples (Table 2). These data confirm that the present method has satisfactory accuracy, precision, and reproducibility for the quantification of efavirenz throughout a wide dynamic range.

**Table 1:** HPLC-MS method for the quantification of nine anti-HIV drugs from dry plasma spot on glass filter and their long term stability in different conditions.

Accuracy and Precision for Calibration Standards of Efavirenz in human plasma (n = 8)			
Nominal Concentration (ng/mL)	(Mean±SD) (ng/mL)	CV (%)	Accuracy (%)
1	0.995±0.111	11.1	100
2.5	2.50±0.259	10.4	100
5	5.14±0.415	8.07	103
10	10.3±0.618	6.01	103
50	51.9±3.30	6.37	104
100	99.6±6.52	6.55	100
250	249±10.9	4.38	100
500	505±24.5	4.85	101
1000	974±71.5	7.34	97.4
2500	2402±181	7.52	96.1

Accuracy and precision for Efavirenz Calibration standards in human plasma.

**Table 2:** Summary of Validation outcomes of Efavirenz in human plasma.

Intra Day and Inter Day Accuracy and Precision for Efavirenz in human plasma							Nominal Conc. (ng/mL)	Recovery (n = 6)	
Nominal Conc. (ng/mL)	Intra Day (n = 6)			Inter Day (n = 3 days)					
	(Mean±SD) (ng/mL)	CV (%)	Accuracy (%)	(Mean±SD) (ng/mL)	CV (%)	Accuracy (%)		Recov. (%)	CV (%)
**1**	1.12±0.103	9.24	112	1.07±0.214	12.3	108	**Internal Standard**	88.6	4.53
**4**	4.42±0.284	6.42	111	4.18±0.383	9.18	104	**4**	95.4	8.22
**80**	86.0±5.12	5.95	107	86.3±4.94	5.72	108	**80**	93.5	4.64
**800**	816±23.5	2.88	102	819±24.8	3.03	102	**800**	102	3.40
**2000**	1993±48.0	2.41	100	1903±103	5.42	95.2	**2000**	107	5.94

### Recovery

At 4, 80, 800, and 2000 ng/mL QC concentrations the percent extraction recoveries at six replicates were 95.4%, 93.5%, 102%, and 107%, respectively. For internal standard percent extraction recoveries was 88.6% (Table 2). Data indicated that the extraction efficiency for efavirenz and internal standard using protein precipitation was satisfactory and was not concentration dependent.

### Assay specificity and ionization suppression (matrix effect)

Analysis of six different blank plasma samples and the corresponding spiked low QC (4 ng/mL) showed no significant interference from endogenous compounds and showed an accuracy of 111%±6.42 at low QC (4 ng/mL, Table 2). Potential interference by concomitantly administered co-drugs antiretroviral drugs to the patients (tenofovir, emtricitabine and ribavirin) was evaluated by spiking blank plasma (n = 6) at their corresponding therapeutic concentration (5 µg/mL) in low QC Efavirenz (4 ng/mL). The difference between the low QC with and without antiretroviral drugs, was calculated, and the result suggested that there was no interference from other antiretroviral agents, in quantification of Efavirenz in human plasma at low QC level (4 ng/mL) employing our method (accuracy: 102%±3.71 for Efavirenz). This is also consistent with previously reported results [23], [28]. Matrix effect can affect the reproducibility from the analyte or the internal standard of the assay. The matrix effect or intensity of ion suppression or enhancement is caused by co-eluting matrix components. The matrix effect of efavirenz was calculated using the following formula: % matrix effect  =  (A/B) ×100%. A represents the peak area response of the analyte in the post-extracted spiked sample and B represents the peak area of pure analyte in the neat solution (50% acetonitrile). A value of >100% indicated ionization enhancement and a value of <100% indicated ionization suppression. The matrix effect was tested at low QC concentration for six individual lots of blank plasma and pooled plasma utilizing previously reported method [29]. Only minor differences were observed between the pure standards and the post-extracted spiked samples (data not shown), illustrating that the HPLC separation conditions had little effect by background signal of plasma after simple protein precipitation clean up step.

### Analyte stability

The stability of efavirenz was investigated to cover expected conditions during sample preparation and storage for all samples, which include stability data from freeze/thaw, short-term (bench top, 24 hrs), and long term (30 day) stability. The precision for freeze/thaw samples ranged from 4.82–10% and the accuracy ranged from 89.3–111%. The results indicated that the analyte was stable in plasma for three cycles when stored at −80°C and thawed to room temperature. The precision for bench-top (24 hours) stability ranged from 4.05–6.59% and the accuracy ranged from 92.8–103% (Table 3). The precision and accuracy for long term stability assessment (30 days) ranged from 4.64–14.3% and 96.4–103%. The precision for autosampler stability ranged from 1.87–9.94%, and accuracy ranged from 99.7–111% at 4°C, showing stability of post extract solvent for 72 hrs at 15°C. Incurred sample reanalysis (data not shown) was performed on a set of samples along with 1 month long term stability, which indicated reliable stability under the experimental conditions of the analytical runs. The stability of the efavirenz and internal standard stock solution was tested and established at room temperature for 4 hr, 24 hr and at –20°C for 30 and 90 days (data not shown). Peak area of efavirenz and internal standard was obtained from fresh stock solution was compared to peak area obtained after 3 months storing at –20°C. Efavirenz and ^13^C_6_-Efavirenz stocks were found to be stable for 3 months at –20°C. The results revealed optimum stability of efavirenz stock solutions for validated assay conditions.

**Table 3:** Summary of stability outcomes of Efavirenz in human plasma.

Nominal Conc. (ng/mL)	Auto-sampler stability 72hr (n = 6) (Mean±SD) (ng/mL)	CV (%)	Accuracy (%)	Stability Bench Top 24hr at RT (n = 6) (Mean±SD) (ng/mL)	CV (%)	Accuracy (%)	Stability 3 cycle freeze (-80°C) thaw (n = 6) (Mean±SD) (ng/mL)	CV (%)	Accuracy(%)	Long term Stability Day 30 (n = 6) (Mean±SD) (ng/mL)	CV (%)	Accuracy (%)
4	4.20±0.418	9.94	105	4.14±0.27	6.59	103	4.43±0.438	9.87	111	3.86±0.553	14.3	96.4
80	88.6±3.74	4.22	111									
800	828±45.8	5.54	103									
2000	1993±37.2	1.87	99.7	1857±75.3	4.05	92.8	1787±86.2	4.82	89.3	2058±95.6	4.64	103

## Discussion

**Figure 3:**
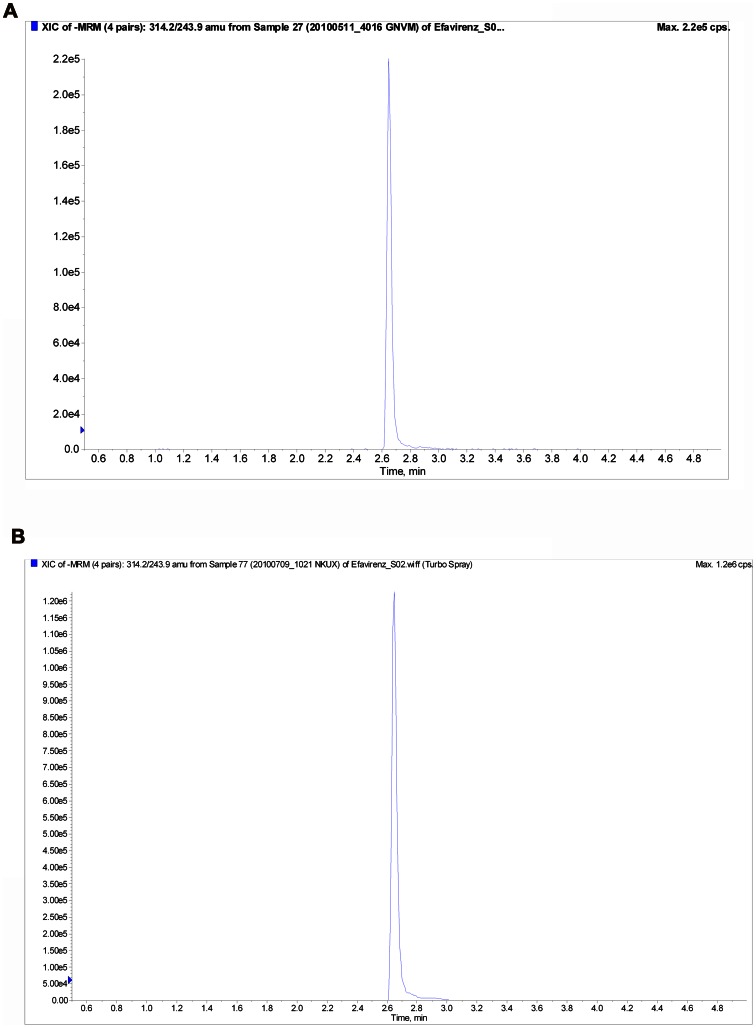
Representative chromatograms of Efavirenz in human plasma clinical study samples with calculated concentrations. A) 20100511_4016 GNVM (244 ng/mL) and B) 20100709_1021 NKUX (2220 ng/mL).

Quantification of drugs in various biological matrices by LC-MS/MS, tandem mass spectrometry techniques are method of choice due to high throughput and increased sensitivity and selectivity, as compared to other spectrophotometric techniques like HPLC-UV, GC-MS and HPLC with fluorescence detection. Various methods are being developed requiring minimal use of biological matrices, so as to have less invasive procedures, and minimal blood collection, which can be the bottleneck, particularly in pediatric studies. We developed an efavirenz assay that has a simple and rapid sample preparation and a selective and sensitive LC-MS/MS method capable of analyzing small volumes of plasma samples. We utilized ^13^C_6_-efavirenz as internal standard which helps compensate matrix effects. Eeckhaut *et al*. [31] showed that labeled internal standard can help to reduce the matrix effect and can compensate for endogenous matrix enhancement and suppression caused by endogenous materials. The LC-MS/MS assay reported here accurately and precisely measures efavirenz concentration in 50 µL plasma specimens with a limit of quantification of 1 ng/mL. The method reported here is simple, has a short run time, and is reliable and rugged. This is the first method, reporting long term stability of efavirenz in human clinical plasma samples. With linearity over wide dynamic range of concentrations, the assay is well suited for use in pharmacokinetic studies. We currently are utilizing this assay to support a University of Pennsylvania human pharmacokinetic study of efavirenz in an African population [30]. Representative chromatograms of efavirenz in human clinical plasma samples are shown in Figure 3A and 3B.

## Conclusion

The LC-MS/MS method described here allowed accurate and reproducible method for determination of efavirenz in human plasma utilizing 50 µL of plasma and short analytical run time of 5 min with an LLOQ of 1.0 ng/mL. High extraction efficiency and low limit of quantification make this method suitable for the use in clinical trials for therapeutic drug monitoring of efavirenz. This method is now successfully applied for routine therapeutic drug monitoring and pharmacokinetics studies in HIV infected patients.
